# Steroid, thiamine, and ascorbic acid during post-resuscitation period for comatose out-of-hospital cardiac arrest survivors (STAR) trial: Protocol for a clinical trial

**DOI:** 10.1371/journal.pone.0319733

**Published:** 2025-04-11

**Authors:** Youn-Jung Kim, Byuk Sung Ko, Young-Il Roh, Yong Hwan Kim, Won Young Kim

**Affiliations:** 1 Department of Emergency Medicine, Asan Medical Center, Ulsan University College of Medicine, Seoul, Korea; 2 Department of Emergency Medicine, College of Medicine, Hanyang University, Seoul, Korea; 3 Department of Emergency Medicine, Yonsei University Wonju College of Medicine, Wonju, Korea; 4 Department of Emergency Medicine, Samsung Changwon Hospital, Sungkyunkwan University School of Medicine, Changwon, Korea; CHU Nantes, FRANCE

## Abstract

**Background:**

Systemic ischemic-reperfusion injury following cardiac arrest results in multisystem organ failure, brain injury and death. The aim of this trial is to investigate whether the combined use of cortisol, ascorbic acid (vitamin C), and thiamine during the early post-resuscitation period reduces the neurologic injury among out-of-hospital cardiac arrest (OHCA) survivors treated with targeted temperature management (TTM).

**Method:**

This is a single-blind, multi-center, randomized, placebo-controlled trial to be conducted in nine tertiary university-affiliated hospitals in South Korea. A total of 160 OHCA survivors treated with TTM will be randomly assigned to the treatment or control groups (1:1 ratio). For the treatment group, patients will intravenously receive a combination dose of ascorbic acid (50 mg/kg, maximum single dose 3 g), thiamine (200 mg), and cortisol (100 mg) that will be mixed in three separate 50mL bags of 0.9% saline, respectively, every 12 hours for 3 days. For the placebo group, patients will receive three separate 50mL bags of 0.9% saline intravenously in the same manner. The primary outcome is the peak neuron-specific enolase level at 48–72 hours after the return of spontaneous circulation.

**Discussion:**

The potential benefits of ascorbic acid, thiamine, and cortisol as neuroprotective agents have been reported in previous preclinical trials. This trial is the first clinical trial to assess the neuroprotective effectiveness of a combination of ascorbic acid, thiamine, and cortisol for OHCA survivors.

**Trial registration:**

ClinicalTrials.gov NCT04921189

## Introduction

Cardiac arrest remains a significant global health challenge, with less than 8% of out-of-hospital cardiac arrest (OHCA) patients achieving favorable recoveries despite advancements in resuscitation medicine [1]. Systemic ischemic/reperfusion injury is a major injury mechanism in OHCA patients, which contributes to multiple organ failure, known as post-cardiac arrest syndrome [2,3]. Proposed mechanisms of injury include mitochondrial dysfunction, increased reactive oxygen species (ROS), metabolic disruption, the inflammatory cascade, and neuronal excitotoxic death, which persist after the return of spontaneous circulation (ROSC) [4,5]. Targeted temperature management (TTM) is the only established therapeutic method during the post-cardiac arrest period for comatose OHCA survivors [6,7]; however, additional pharmacological interventions to reduce the injuries could be beneficial.

Ascorbic acid (vitamin C), thiamine, and corticosteroids are known as potential therapeutic agents to alleviate ischemic/reperfusion injury after cardiac arrest [5]. Ascorbic acid acts as an antioxidant defense substance, reducing ROS and reactive nitrogen species and improving microcirculation by limiting oxidative injury and endothelial barrier disruption [8]. Thiamine is a key cofactor needed for pyruvate dehydrogenase function to resume aerobic metabolism and, therefore, to recover from mitochondrial dysfunction [9]. Corticosteroids can decrease the inflammatory response and improve microcirculatory flow [4,10]. It is also helpful to maintain hemodynamic stability for patients with post-arrest hypotension by enhancing vasopressor effects [11]. Theoretical and experimental studies suggested that the concomitant use of cortisol, thiamine, and ascorbic acid might have potential treatment synergism for whole-body ischemia/reperfusion injuries after cardiac arrest [12–14].

Many preclinical studies reported the beneficial effects of singular drugs such as thiamine, ascorbic acid, and corticosteroids in cardiac arrest animal models [9,15–17]; however, the limited human clinical trials have failed to show benefits [11,18]. The simultaneous occurrence of many dysregulated pathways during the post-cardiac arrest period necessitates the use of medications that can reduce the cascades of ischemic-reperfusion injury and successfully target the intended route [4]. The aim of this trial is to investigate whether the combined use of cortisol, ascorbic acid, and thiamine during the early post-resuscitation period would reduce neurologic injury among the OHCA survivors treated with TTM.

## Materials and methods

### Study design

**Fig 1:**
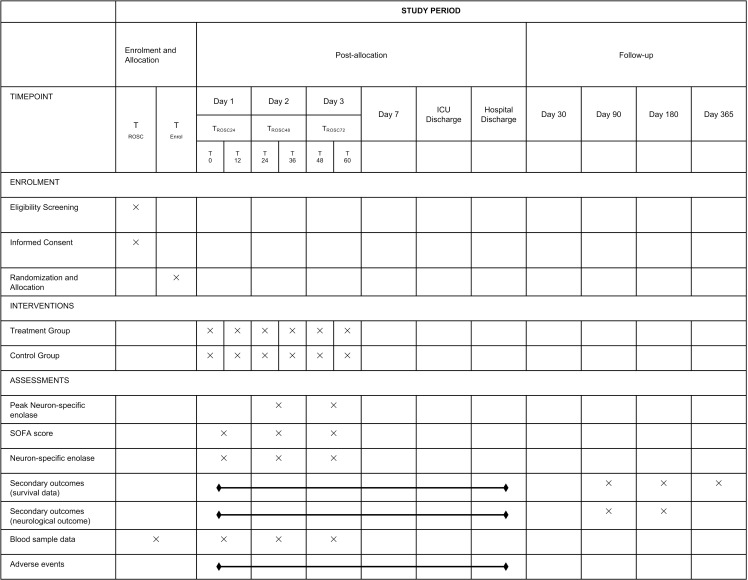
Data collection and follow-up of the participants in the STAR trial (SPIRIT figure). ROSC = Return of spontaneous circulation; SOFA = sequential organ failure assessment.

**Fig 2:**
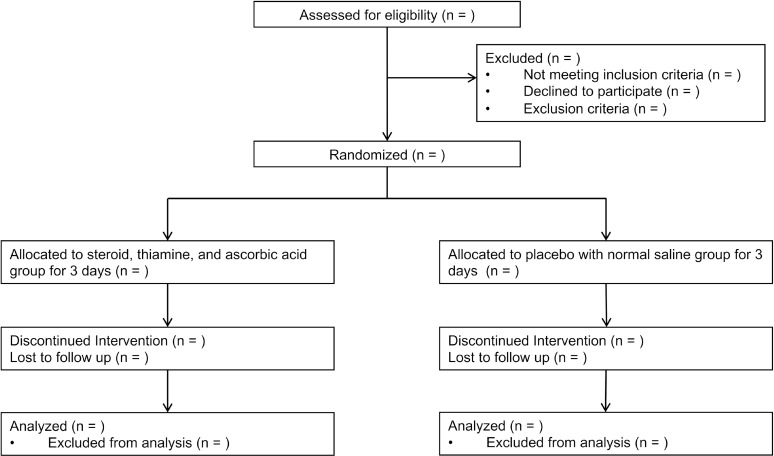
Flow chart of the study protocol.

The steroid, thiamine, and ascorbic acid during the post-resuscitation period for comatose OHCA survivors (STAR) trial is a single-blind, multicenter, randomized placebo-controlled trial being conducted in nine emergency departments (EDs) of tertiary university-affiliated hospitals in South Korea. The institutional review board of all participating hospitals have reviewed and approved the study protocol, including the institutional review board of Asan Medical Center (No. 2021–0613, approved on April 27, 2021). The STAR trial is registered under clinicaltrials.gov as protocol NCT04921189 on June 4, 2021. The study started on December 31, 2021 and the estimated study completion date is June 5, 2025. The investigators have obtained written informed consent from all patients’ legal surrogates. The Ministry of Food and Drug Safety in Korea has also approved the trial protocol. All OHCA survivors are being screened for eligibility by the attending emergency physician. Fig 1 shows the study schedule and Fig 2 illustrates the study flow chart.

### Population

The study population will consist of adult patients (19–79 years old) with successfully resuscitated OHCA treated with TTM (target temperature between 32 and 36 °C). The patients over 79 years old were excluded due to the increased risk of comorbidities and frailty, which could confound the interpretation of neuroprotective effects and outcomes. The participating patients must meet all the inclusion criteria and none of the exclusion criteria (Table 1).

**Table 1:** Inclusion and exclusion criteria.

Inclusion criteria	Exclusion criteria
An out-of-hospital cardiac arrest survivors treated with targeted temperature management (target between 32 and 36 °C)	Patients who > 12 hours from cardiac arrest to drug or placebo administration
Cardiac arrest without obvious non-cardiac cause	Patients who have set limitations on treatment (e.g., patients with a signed do-not-resuscitate order), or with an underlying terminal-stage disease without an active treatment plan and those who are not expected to survive to discharge, or who are expected to die within 24 h despite best possible treatment, based on the judgement of medical personnel
	Patients taking at least 1 g/day of vitamin C or receiving intravenous thiamine prior to enrolment
	Known poor neurological status (Cerebral Performance Categories 3–5); glucose-6-phosphate dehydrogenase deficiency; pregnancy; thalassemia; hyperoxaluria; cystinuria; or ongoing gout attacks
	History of previous cardiac arrest, or oxalate renal stones
	Patients who do not voluntarily consent to participate in the trial (by legal proxy)

### Randomization, treatment allocation, and blinding

The patients will be randomly assigned in a 1:1 ratio to either the treatment group or the placebo group. The randomization sequence will be generated by *R* (version 3.3.3; R Foundation for Statistical Computing, Vienna, Austria; https://www.R-project.org) with randomized blocks of 4, stratified by recruitment site. The allocation sequence will be concealed from the attending emergency physicians and researchers enrolling and assessing patients in sequentially numbered, opaque, sealed, and stapled envelopes. The envelopes will be opened only at the allocation time. Given that patients are in a comatose state, they cannot perceive or differentiate the treatment solution from the placebo. To ensure blinding, study medications and placebo solutions are prepared by independent, unblinded personnel who are not involved in patient care or outcome assessments. However, the research team responsible for patient care and administering the treatments is aware of the group allocation. The primary outcome of this study, peak NSE levels at 48 and 72 hours after ROSC, is an objective biomarker, reducing the risk of bias associated with unblinded personnel. Additionally, secondary outcomes will be assessed by blinded evaluators to further minimize potential bias.

### Interventions

As soon as possible after achieving ROSC at the ED or arrival after achieving ROSC before ED admission, patients who meet inclusion criteria will be identified. After written consent is obtained from legal surrogates, a unique randomization number will be allocated to each participant in the order of enrolment. For the treatment group, ascorbic acid (50 mg/kg, maximum single dose 3 g, daily dose 6 g), thiamine (200 mg, daily dose 400 mg), and corticosteroid (100 mg, daily dose 200 mg) will be mixed in three separate 50mL bags of 0.9% saline, respectively, and administered through intravenous infusion over 60 min every 12 h for 3 days. For the placebo group, an identical volume of 0.9% saline (50 mL, respectively) will be administered through intravenous infusion over 60 min every 12 h for 3 days. The first administration of the study medication is aimed as soon as possible after ROSC, at least not to delay over 12 hours after ROSC.

All the participants will be treated with post-resuscitation care in accordance with the current guidelines [6,19,20]. Depending on the attending emergency physicians’ decision, in case of clinically suspected vitamin C, thiamine, or adrenal insufficiency, supplementation of ascorbic acid, thiamine, or corticosteroids is permitted. For patients requiring high dose vasopressors, corticosteroid administration is permitted based on the attending physician’s judgment. If the legal proxy requests withdrawal from the trial, the collected data and blood samples will be removed.

### Data collection

T_ROSC24_, T_ROSC48_, and T_ROSC72_ indicate the time points of 24, 48, and 72 hours after ROSC, respectively. T_0_ is the time point of the first drug administration, which will aim to be administered within 12 hours after ROSC. The patients will receive the medication every 12 hours after T_0_, six times. T_ROSC24_ is between T_12_ and T_24._ Blood samples for plasma ascorbic acid and thiamine levels will be collected at enrolment prior to study drug administration and 72 hours after ROSC which will be transferred to the central laboratory for analysis. Blood samples for neuron-specific enolase (NSE) levels will be collected at 24, 48, and 72 hours after ROSC, and analyzed at the local laboratory of each participating hospital. A laboratory medicine specialist in all participating academic hospitals will assess the hemolysis index before reporting NSE values to ensure accurate NSE levels. Other blood samples will be collected at enrolment prior to study drug administration and daily for 3 days. We plan to collect and store serum samples at the same time, which will be frozen at -80°C immediately for future biomarker analysis. Adverse events and survival data will be monitored daily during hospitalization. The investigators will be blinded to the study group assignments and will conduct follow-up telephonic interviews for survival and neurocognitive function after hospital discharge around days 30, 90, 180, and 365.

An independent monitoring officer at the coordinating center will monitor the study procedures and data quality according to the Korea Good Clinical Practice guidelines in all participating centers. All data will be anonymized and collected using standard clinical report forms by investigators at each participating hospital. The monitoring officer will review and verify all data from inclusion to follow-up. The data will be stored at the coordinating center.

### Outcome measure

The primary outcome will be the peak NSE level at 48 or 72 hours after ROSC. If a patient dies before 48 hours, the patient will be excluded from the primary analysis. If a patient dies between 48 and 72 hours after ROSC, the NSE level at 48 hours will be used. Secondary outcomes will include as follows: ΔNSE level, Δ sequential organ failure assessment (SOFA) score, mortality (7-day, 30-day, 90-day, 180-day, in-hospital), time to death (days), length of intensive care unit (ICU) stays (days, up to 1 year), length of hospital stays (days, up to 1 year), ICU-free days within 14 days, time to awakening (days to neurological recovery, defined as Glasgow coma scale >13 after ROSC) and neurological outcomes (30-day, 90-day, 180-day). The ΔNSE level is the serial change in NSE level that reflects the severity of reperfusion injury during the post-cardiac arrest period (ΔNSE level = the peak NSE level at 48 and 72 hours after ROSC NSE level at 24 hours after ROSC). The Δ SOFA score is defined as the difference between the SOFA score at enrolment and the maximum SOFA score between 24 and 72 hours after ROSC (ΔSOFA score = initial SOFA score at enrolment − maximum SOFA score between 24 and 72 hours after ROSC). If a patient dies in the first 72 hours, the maximum SOFA score will be counted as 24. Neurological outcomes will be assessed using cerebral performance category scores and the modified Rankin scale at 30, 90, and 180 days after ROSC. A good neurologic outcome is defined as cerebral performance category scores of 1–2 and modified Rankin scale of 0–2, respectively.

### Statistical analyses

All statistical analyses will be performed with the intention-to-treat set, the full analysis set and the per-protocol analysis set. The primary analysis will exclude patients who die within 48 hours after ROSC, missing data on serum NSE level at 48 hours and 72 hours, consistent with the study design and sample size calculation. Supplementary analyses will include all randomized patients, and missing data will be handled using multiple imputation techniques to address potential bias. A multiple imputation approach will generate multiple datasets with imputed values, which will then be analyzed using the same statistical methods described for the primary and secondary outcomes. The results will be compared with those from the complete-case analysis to ensure robustness.

Primary and secondary outcomes will be analyzed using mixed-effects models, with recruitment site included as a random effect to account for between-center variability. Covariates such as age, sex, initial arrest rhythm, total collapse time, underlying disease, and grade of revised post-Cardiac Arrest Syndrome for Therapeutic hypothermia score [21] will be included as fixed effects, particularly if imbalances are observed. Mixed-effects models provide a more conservative and reliable approach than simple t-tests, allowing for appropriate handling of multi-center data. Survival duration and time to awakening will be assessed by the Kaplan–Meier method and compared by the log-rank test. Subgroup analysis will be conducted by predefined groups according to age, sex, initial arrest rhythm, arrest cause, total collapse time, NSE level at 48 hours, grade of revised post-Cardiac Arrest Syndrome for Therapeutic hypothermia score, timing of first medication administration, vitamin C level, and thiamine level. Two-tailed P-values less than 0.05 will be considered statistically significant. Statistical analysis will be performed using SAS version 9.4 (SAS Institute, Cary, NC, USA), IBM SPSS for Windows, version 21.0 (IBM Corp., Armonk, NY, USA), and R (version 3.6.1; R Foundation for Statistical Computing, Vienna, Austria; https://www.R-project.org).

### Sample size calculation

Based on Korea prospective multicenter registry cohort data, post-cardiac arrest survivors without obvious non-cardiac cause treated with TTM had a mean peak NSE level of 114 and a standard deviation of 119 [Unpublished]. Hypothesizing a 50% reduction in the treatment group and no change in the placebo group, we calculated that 68 patients would be needed per group with a power of 80% and alpha level of 0.05. Assuming a dropout rate of 15% due to potential death within 48 hours after ROSC, missing data on serum NSE level at 48 hours and 72 hours, and follow-up loss, we determined that 80 patients would be included in each group. The recent European Resuscitation Council/European Society of Intensive Care Medicine (ERC/ESICM) guidelines recommend that an NSE threshold level >60 ng/mL at 48 and/or 72 hours after cardiac arrest indicates poor outcomes in their prognostication strategy algorithm [6]. To observe a clinically meaningful improvement in neurological outcomes, a substantial reduction in NSE levels is necessary to approach the guideline’s cutoff for favorable prognosis.

### Data and safety monitoring

An independent data safety monitoring board consisting of two independent members (an intensivist and an emergency physician) will evaluate safety during the trial annually without any interim analysis. Adverse events, including urticaria or rash, angioedema or anaphylaxis, vomiting, diarrhea, gastrointestinal bleeding, ventilator-associated pneumonia, catheter-related infection, and uncontrolled hyperglycemia, will be recorded both prospectively and by review of clinical charts. All institutions participating in this trial are also part of the Korean Hypothermia Network Prospective Registry (KORHN-PRO; NCT02827422), which includes standardized protocols for blood glucose monitoring, measured from arterial or venous samples after ROSC and every 4 hours from the initiation of TTM to 72 hours. As capillary blood glucose devices are not utilized in this clinical protocol, the issue of factitious hyperglycemia induced by high-dose intravenous vitamin C was not considered a concern in this trial. All serious adverse events will be reported to the Institutional Review Boards of the individual participating hospitals within seven days of the investigator being made aware of the event. All suspected unexpected serious adverse reactions will be reported to the Institutional Review Boards of the individual participating hospitals, the coordinating center, and the Ministry of Food and Drug Safety in Korea within seven days of the investigator being made aware of the event.

### Funding and support

The STAR trial is supported by the Korean Hypothermia Network (KORHN). This research was supported by a grant of the Korea Health Technology R&D Project through the Korea Health Industry Development Institute (KHIDI), funded by the Ministry of Health & Welfare, Republic of Korea (grant number: RS-2024–00335934) and the National Research Foundation of Korea (grant number: NRF‑ 2021R1A2C2014304). The funders had no role in the design and conduct of the study; collection, management, analysis, and interpretation of the data; preparation, review, and approval of the manuscript; or the decision to submit the manuscript for publication.

### SPIRIT checklist

The trial design follows the Standard Protocol Items Recommendations for Interventional Trials (SPIRIT). The SPIRIT Checklist is presented in S1 File.

## Discussion

The STAR trial is the first single-blind, multi-center, randomized placebo-controlled trial to compare the neuroprotective effects of early extensive multi-drug combination therapy, including high-dose ascorbic acid, thiamine, and corticosteroids in comatose OHCA survivors treated with TTM. Despite advances in resuscitation techniques and intensive care, treatment of significant neurological deficits in OHCA survivors is challenging; moreover, most clinical trial interventions for OHCA have failed to improve outcomes. The clinical trials for evaluating the effect of pharmacological treatment in cardiac arrest patients remain limited and did not show beneficial effects [18,22,23].

The optimal dose, administration timing, and the length of supplementation are crucial factors for pharmacological efficacy. The systemic injury after cardiac arrest constitutes a multiphase process that can occur over days, including excitotoxicity, mitochondrial dysfunction, oxidative stress, and inflammation [5,24]. In our recently published before-after study, high-dose intravenous administration of vitamin C and thiamine did not demonstrate significant improvement in one-month neurological outcomes for OHCA patients treated with TTM [25]. Despite the inconclusive results, older patients aged 65 years or more showed significantly improved neurologic outcomes (adjusted OR, 5.53; 95% CI, 1.21–25.23; P = 0.03), suggesting the potential benefits of a personalized approach [25]. Additionally, the study focused solely on vitamin C and thiamine [25], whereas the STAR trial incorporates hydrocortisone to explore the synergistic effects of a multi-drug combination. Given that complex pathophysiology, we planned to administer ascorbic acid, thiamine, and corticosteroids as multiple pathway-specific agents for three days, generally considered the period of active reperfusion injury [5]. Those drugs can attenuate whole-body ischemia/reperfusion injuries especially brain injuries after cardiac arrest during the first 72 hours after ROSC [4,5]. Previous clinical trials for evaluating exogenous vitamin C as a neuroprotective agent in acute neurological injury administered the median daily dose of vitamin C ~ 10 mg/kg, approximately one-tenth of the dose used in animal studies [26]. To accomplish the supraphysiological plasma concentrations for attenuating the burst of ROS generation, we chose 50 mg/kg of ascorbic acid (maximum single dose 3g) every 12 hours, total maximum dose 6 g a day. The requirement dose of thiamine in OHCA patients has not been elucidated [9]; however, a recent clinical trial in sepsis and septic shock demonstrated that 200 mg of thiamine every 12 hours for 3 days was sufficient to normalize the thiamine levels [27]. Considering the pathophysiologic similarity between postcardiac arrest syndrome and septic shock, we chose 200 mg of thiamine every 12 hours. The dose of corticosteroids was chosen as 100 mg every 12 hours, which is in line with the consensus guidelines of the American College of Critical Care Medicine and its theoretical synergistic benefit [28,29].

Several ongoing randomized controlled trials are exploring the effects of single-drug therapy in OHCA patients, primarily targeting organ failure and shock reversal. The VITaCCA (early high-dose vitamin C in post-cardiac arrest syndrome, NCT03509662) trial investigates the impact of supplementation (3 g/day) and pharmacological (10 g/day) doses of vitamin C on organ dysfunction over 96 hours in OHCA patients [30]. Similarly, the VICEPAC (early intravenous high-dose vitamin C in postcardiac arrest shock, NCT05817851) trial aims to assess the ability of high-dose intravenous vitamin C in improving shock reversal [31]. The HYVAPRESS (hydrocortisone and vasopressin in post-resuscitation syndrome, NCT04591990) trial investigates the superiority of arginine-vasopressin and hydrocortisone over norepinephrine in improving survival and neurological outcomes in OHCA patients with post-resuscitation shock, without specifying mental status as part of the inclusion criteria. In contrast, the STAR trial uniquely targets comatose OHCA survivors and aims to evaluate the neuroprotective effects of a multi-drug combination of vitamin C, thiamine, and hydrocortisone.

We chose the peak NSE level at 48 and 72 hours as the primary outcome. Serum NSE is well correlated with neuronal cell damage and hypoxic-ischemic brain injury, and it is the only recommended biomarker for multimodal prognostication in the American Heart Association and European Resuscitation Council/European Society of Intensive Care Medicine’s guidelines [6,19]. However, NSE levels can be falsely elevated due to several medical conditions, including neuroendocrine tumors, small cell lung cancer, and the use of medical devices that can cause hemolysis, such as extracorporeal membrane oxygenation (ECMO), hemodialysis, and intra-aortic balloon pumps. To address this, we have planned to collect detailed clinical data on comorbidities and the use of ECMO and hemodialysis. Additionally, a laboratory medicine specialist in all participating academic hospitals will assess the hemolysis index before reporting NSE values to ensure accurate NSE levels. The peak NSE level is a reasonable approach to assess the neuroprotective effects of high-dose ascorbic acid, thiamine, and corticosteroids in comatose OHCA survivors. The assumed 50% reduction in NSE levels may overestimate the treatment effect, and smaller effects might not be detectable with the calculated sample size. The interpretation of the findings would be a potential limitation of the study. Also, this study is primarily powered for the primary outcome. Given the limited sample size, secondary outcomes analyses may lack sufficient power to detect smaller effects. As such, these results will be interpreted cautiously and considered exploratory, providing a basis for future studies.

In conclusion, this study will investigate the effects of high-dose ascorbic acid, thiamine, and corticosteroids in comatose OHCA survivors treated with TTM on neurological injury using peak NSE concentrations. This will provide preliminary information about future neuroprotective combination therapy for OHCA survivors.

### Trial status

The first patient was included on December 31, 2021. Recruitment is active. The estimated study completion date is June 2025.

## Supporting information

S1 File SPIRIT-Checklist. (DOCX)
